# The Diagnosis, Management and Complications Associated with Fractures of the Talus

**DOI:** 10.2174/1874325001711010460

**Published:** 2017-05-31

**Authors:** Barnett J.R, Ahmad MA, Khan W, O’ Gorman A.

**Affiliations:** 1 Royal National Orthopaedic Hospital, Stanmore, Middlesex, HA7 4LP, UK; 2 Department of Trauma and Orthopaedics, Addenbrooke's Hospital, Cambridge, CB2 0QQ, UK; 3 Department of Trauma and Orthopaedics, The Whittington Hospital, Magdala Ave, London, N19 5NF, UK

**Keywords:** Talus, Fractures, Blood supply, Talar neck fractures, Complications

## Abstract

Fractures of the talus are uncommon injuries that can have devastating consequences. The blood supply to the talus is tenuous and injuries are associated with a higher incidence of avascular necrosis and malunion. Talar neck fractures are the most common fractures. This review looks at the different types of fractures of the talus, particularly focusing on talar neck fractures. The diagnosis and management are discussed as well as the complications.

## 1 INTRODUCTION

Fractures of the talus are uncommon, accounting for approximately 0.1% of all fractures. The talus is the second most common tarsal bone to be injured after the calcaneus [1]. Fractures of the talus are associated with significant morbidity in view of the blood supply, and these can be challenging for surgeons to manage [2]. The talus is a dense bone and fractures in non-pathological bone usually result from high energy impacts e.g. motor vehicle collisions or a fall from a significant height [3]. Fractures of the talus were first described in parachutists and pilots of the Royal Air Force who sustained these injuries upon impact with the ground, hence termed aviator’s astragalus [4, 5].

Fractures of the talus can be classified by their anatomical locations, and include talar neck, head, body, lateral process and posterior process fractures. Of these fractures, talar neck fractures are the most common. Elgafy *et al* (2000) found that talar neck fractures constituted 45% of talar fractures in their study of 60 patients [6]. Snowboarders are at increased risk of lateral process fractures due to the forces involved in landing a jump and the position of the feet when hitting the ground [7].

The results with surgical management of these fractures have improved over the last few years, largely due to a greater understanding of the anatomy of the region [8]. Optimal reduction often necessitates both anteromedial and anterolateral exposures during reduction and fixation [8]. This method of reduction and fixation is a possible explanation for reduced morbidity and better results. In this review paper, we describe the anatomy and clinical presentation of these injuries.

## 2 ANATOMY

The talus is the tarsal bone that articulates with the tibia and fibula to form the ankle joint. Its biomechanical role includes the transmission of forces between the lower leg and the foot. It does not have any musculotendinous attachments [2].

The talus is anatomically composed of three parts, the body, neck and head. Fig. (**1a**) shows the superior view of the talus and Fig. (**1b**) shows the inferior view. The talar body articulates with the tibia superiorly at the ankle joint, and with the calcaneus inferiorly at the subtalar joint. The talar head articulates with the navicular anteriorly. The talar neck, between the head and the body, faces anteromedially [2] and forms the weakest part of the bone [3]. Fig. (**1**) shows that the talus is widest at the anterior aspect, limiting rotational movements when the foot is dorsiflexed and conferring greater stability. In dorsiflexion, the talar dome forces the fibula laterally into external rotation. In plantar flexion the ankle’s ligamentous attachments provide joint stability. These include the deltoid ligament medially and the lateral collateral ligaments of the ankle laterally [2].

The blood supply to the talus was described by Wildenauer and then by Haliburton in the 1950s [9]. The arteries that supply the talus are the posterior tibial, the anterior tibial and the perforating peroneal arteries [1]. There is an anastomotic ring that surrounds the talar head and neck and supplies the talar body. Almost 60% of the talus is covered with articular cartilage, and only a small portion of the talus may be perforated by blood vessels. This makes it more prone to avascular necrosis [3]. Additionally where there is a talar neck fracture, the intraosseus branches and artery to the tarsal canal (a branch of the posterior tibial artery) are disrupted. The talar body then only receives blood from the deltoid branch of the posterior tibial artery. This usually supplies the talar body posteromedially and therefore the risk of avascular necrosis is increased [10].

## 3 TALAR NECK FRACTURES

These are the most common fracture of the talus, accounting for approximately half of all talar fractures [11]. The consequences of a talar neck fracture may be devastating due to the significant complications that can arise following this injury. These include avascular necrosis, mal-union and arthritis [12]. Halvorsen **et al** in a systemic review of 848 patients with talar neck fractures reported a 33% incidence of avascular necrosis, 17% incidence of mal-union and 70% cases resulted in a post-traumatic arthrosis [12].

### 3.1 Mechanism of Injury

Talar neck fractures typically result from high energy trauma such as motor vehicle collisions. The mechanism is usually forced dorsiflexion of the foot as the talar neck is forced against the tibial crest [5]. This may occur when the driver exerts pressure on the brake pedal during a collision [5]. This was the mechanism described by Anderson where pilots exerted pressure on the rudder pedal as they crashed to the ground [5, 11]. A caderveric study by Funk **et al** simulated this mechanism of injury. A pedal was driven towards the knee causing dorsiflexion and axial loading. They reported both talar neck fractures and medial malleolar fractures occurring [13].

### 3.2 Classification

Hawkins described a classification for these factures in 1970 which remains in use today [14, 15]. The classification uses plain radiography to assess the severity of the fracture to the talar neck. Fig. (**2**). Type 1 fracture is non-displaced. Type 2 has displacement of the fracture with subluxation/dislocation of the subtalar joint. Type 3 is similar to type 2, with additional subluxation/dislocation of the ankle joint [15]. Type 4 is a modification later added to the Hawkins classification and includes type 3 with talonavicular dislocation [16]. Type II fractures have been further divided by Vallier **et al** into IIa and IIb. The IIa fracture does not include subtalar dislocation whereas IIb does. They observed an increased rate of avascular necrosis where the subtalar joint was dislocated [17].

Halvorsen **et al** in their systematic review showed an increasing trend of avascular necrosis with each Hawkins type. This was 5.7% for Hawkins type 1, 18.4% for Hawkins type 2 and 44.7% for Hawkins type 3. This percentage drops to 12% for type 4 fractures which the authors felt may have been influenced by the rarity of the injury, there were only 33 cases in 848 patients [12].

### 3.3 Clinical Presentation

Due to the high energy required to fracture the talus, these fractures may be part of a multi-trauma presentation. There may be significant deformity necessitating immediate reduction to prevent skin necrosis [1].

The injury tends to cause pain and swelling to the ankle with the inability to bear weight. Examination will usually reveal tenderness in the ankle region with a reduced range of movement. The talar head and neck may be palpated anterior and inferior to the ankle joint. The body may be palpated distal to the malleoli and anterior to the Achilles tendon [18]. As with all orthopaedic examinations assessing the neurovascular status is essential and any deficit will require urgent intervention.

### 3.4 Imaging

Imaging is initially undertaken with plain radiography. The views required are anterior-posterior, lateral and mortise. Plain radiographs may detect talar neck fractures but there is a high false negative rate. A study of 132 talar fractures found that 93% had additional fracture information on Computerised Tomography (CT) scanning that was not found on initial plain radiography [19]. Canale **et al*.* advise special talar views that involve taking oblique x-rays of the foot to further evaluate the talar neck. This shows the calcaneus below the talar head and neck. The ankle is fully plantar flexed with pronation of the foot at 15 degrees. The inferior aspect of the foot is placed on the x-ray table. X-rays are then projected at a 75 degree angle to the table [16].

Further characterisation of the fracture may be carried out with CT scanning. Where a fracture is suspected or confirmed on X-ray, Fig. (**3**), a CT scan should be obtained. A missed talar fracture, even with minimal displacement may have severe long term consequences [1]. A study by Rodop **et al** found that 39% of ankle and midfoot fractures may be missed at this initial stage. In their small study, they found eight cases that were missed initially on plain radiography. These patients were at first treated conservatively. They were eventually diagnosed through Magnetic Resonance Imaging (MRI) and CT scanning [21]. They concluded that if there remains suspicion about a talar fracture then further imaging is recommended to reduce the risk of complications. These imaging modalities may also be beneficial for operative planning [22].

MRI scanning is not usually used in the early stage. It tends to be used where patients continue to complain of symptoms 4-6 weeks following injury in the presence of normal radiographs. It may then be useful to evaluate soft tissues, articular surfaces and possible bony injury [23].

### 3.5 Management

Fractures of the talus are high energy injuries and the patient may often present as a polytrauma with life or limb threatening injuries. In some situations, life and limb saving treatments take priority, but as soon as is safe to do so, this injury should be assessed and managed. Treatment of fractures of the talus depends on the location of the fracture [1]. Where there is an open fracture, neurovascular deficit or dislocation, immediate treatment is required. An undisplaced talar neck fracture where there is adequate alignment of articular surfaces may be treated non-operatively. A cautious approach is required as even minimal displacement may require surgical fixation to avoid complications [1]. Type II-IV fractures require immediate fixation in order to reduce the incidence of complications. This is by open reduction and internal fixation [16]. A French multicentre study of 114 patients looked at internal fixation of both talar neck and body fractures with a five-year follow-up. They found that reduction quality was better with K-wire fixation than screws using the Kitaoka score. Screws could cause excessive compression, especially where comminution was present [22]. They recommended using screws for simple fractures and plates where there is comminution.

Dealing with the complications after a fracture of the talus may necessitate further procedures. In the study by Canale, 25% of patients who had avascular necrosis, malunion, arthritis and infection required a further procedure [16].

In order to identify avascular necrosis of the talus, Hawkins sign may be used. This is seen as a radiolucent band on a radiograph 6-8 weeks following a fracture. Its presence indicates subchondral atrophy and that the talus is unlikely to develop avascular necrosis as there is sufficient vascularity. It has been shown by Tezvel **et al** in a study of 26 patients to have a sensitivity of 100% and a specificity of 57.7% [24]. In those who have undergone avascular necrosis of the talus a number of options are available. These include talectomy, pantalar arthrodesis, tibiotalar arthrodesis and tibiocalcaneal arthrodesis. Dennison **et al** report good functional outcome following excision of the necrotic body of the talus with tibiocalcaneal fusion using an Ilizarov frame [25].

## 4 TALAR BODY FRACTURES

These fractures account for approximately 20% of all fractures of the talus [6]. Fracture of the talar body is not an isolated phenomenon; Sneppen & Buhl [26] found that forces that act upon the talus and result in fractures also affect, and sometimes fracture, the ankle joint. Fractures of the talar body can be classified as compression injuries, shear fractures, fracture of the posterior process, fracture of the lateral process and crush fractures. Talar body fractures have a similar mechanism of injury as talar neck fractures. They tend to occur following axial loading on the ankle through the leg although the foot is not dorsiflexed. They usually involve the articular surfaces, primarily the ankle joint and occasionally the subtalar joint. This increases the likelihood of developing osteoarthritis [27].

These fractures are nearly always managed operatively. Despite this, there is a high rate of complications [8]. These patients may develop osteoarthritis. Vallier **et al** found that 17 of 26 patients developed osteoarthritis of the tibiotalar joint, and nine of 26 patients developed osteoarthritis of the subtalar joint (8). There is a high incidence of disruption to the blood supply and the rate of avascular necrosis has been reported at 25% [27].

## 5 TALAR HEAD FRACTURES

The talar head is the least common part of the talus to fracture. In 228 patients with talar fractures, only 2.6% had a fracture of the talar head [4]. They are usually sustained when the foot is plantar flexed and there is a forced axial load. They tend to be intra-articular injuries and may extend to the neck and body. They are associated with dislocation or subluxation of the talus [1]. Long **et al** describe two patient groups. Older osteoporotic patients who undergo an insufficiency fracture with minimal activity and younger patients sustaining high-energy injuries [28]. The talar head receives a good blood supply that makes it less likely to undergo avascular necrosis than talar neck or body fractures [27].

Talar head fractures may be managed non-operatively or operatively. The aim of treatment is to preserve the articular surfaces and ensure stability of the talonavicular joint. A non-displaced and isolated impaction fracture or an avulsion fracture with no extension into the subtalar joint with minimal disruption of the talonavicular surface can be managed conservatively. For all other talar head fractures, operative fixation is advised [18].

## 6 LATERAL PROCESS FRACTURES

The lateral process of the talus is a wedge shaped prominence that protrudes from the body of the talus. It forms part of the subtalar joint as it articulates with the fibula laterally and the os calcis inferiorly [29]. Fractures of the lateral process are common in snowboarders. They have been shown to account for 15% of ankle injuries in snowboarders due to the impact of landing from jumps [30]. The mechanism of injury involves inversion and dorsiflexion of the ankle whilst applying an axial load [7, 31]. They may be difficult to diagnose on plane x-ray and clinically can appear similar to an ankle sprain. CT scanning is advised where there is concern [32].

The McRory-Bladin classification describes three types of fracture [31]. Type I fractures do not involve articular surfaces. Type 2 fractures involve the joint surface of the ankle and the subtalar joint. Type 3 fractures involve comminution.

Management may be non-operative or operative. Where a fracture is non-displaced and small, it may be managed with cast immobilisation. Larger fractures and displaced fractures are treated with open reduction and internal fixation [33, 34]. Lateral process fractures may be complicated by osteoarthritis and malunion. Koch **et al** found that 45% of patients with this injury were associated with subtalar osteoarthritis [34].

## CONCLUSION

Talar fractures are infrequent but potentially devastating injuries. Talar neck fractures are by far the most common, accounting for 50% of these injuries, and are classified using Hawkins classification. The vascular supply of the talus is such that fractures to the neck and body have a high likelihood of developing avascular necrosis. Other complications of talar fractures include non-union, malunion and osteoarthritis. Operative fixation is used in most cases of talar fractures. Treatment should be expedited where there is evidence of neurovascular deficit or open injury.

## Figures and Tables

**Fig. (1):**
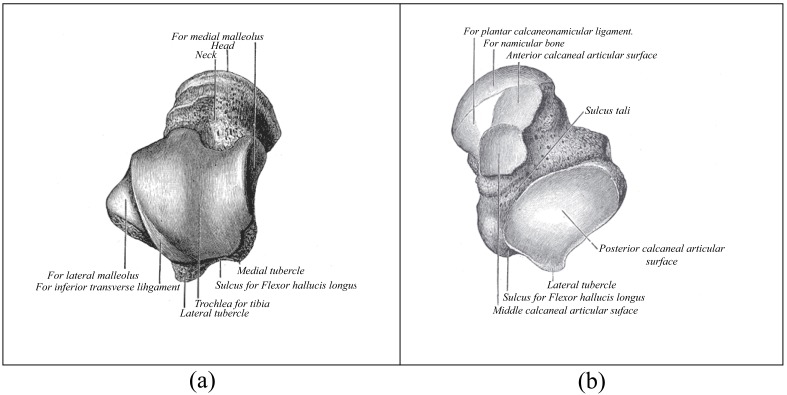
Anatomy of the talus with (a) a superior view, and (b) an inferior view.

**Fig. (2):**
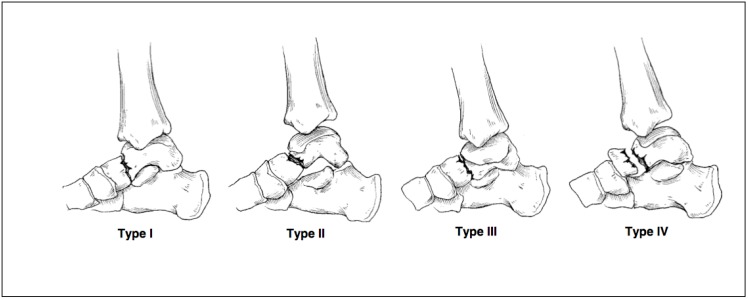
Diagrammatic representation of talar neck fractures by Fortin **et al** (1).

**Fig. (3):**
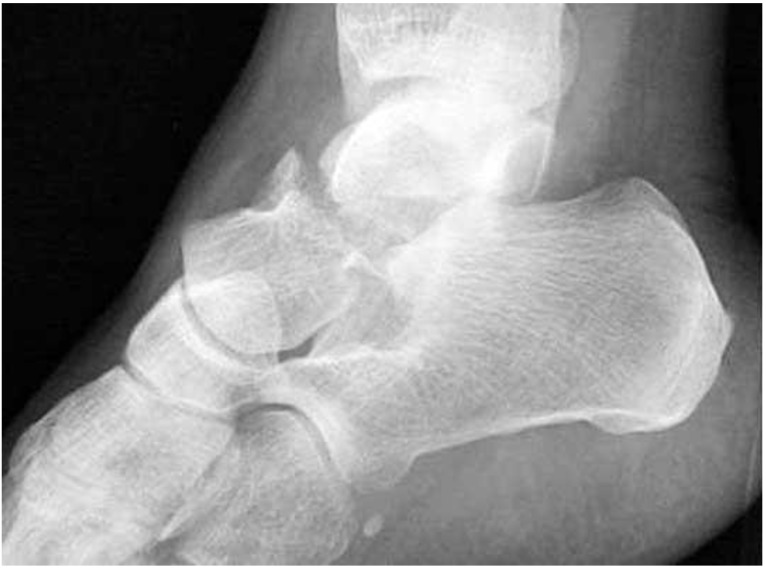
Fracture to the talar neck [20].
